# A Rare Case of Isolated Primary Leiomyoma Discovered Within an Ovarian Endometrioma: A Case Report

**DOI:** 10.7759/cureus.93854

**Published:** 2025-10-05

**Authors:** Michelle Mei Ying Tiong, Sangeeta Mantoo, Shau Khng Jason Lim

**Affiliations:** 1 Department of Obstetrics and Gynaecology, Singapore General Hospital/SingHealth, Singapore, SGP; 2 Department of Anatomical Pathology, Singapore General Hospital/SingHealth, Singapore, SGP

**Keywords:** endometriosis, endometriotic cyst, ovarian endometrioma, ovarian leiomyoma, primary leiomyoma

## Abstract

A woman in her 30s presented to the emergency department with a one-day history of acute lower abdominal pain, which was not related to her menstruation and not associated with any bowel or urinary tract symptoms. Examination findings revealed a palpable pelvic mass extending up to the level of the umbilicus with generalized tenderness and abdominal guarding over the suprapubic and left lower abdominal regions. Transvaginal ultrasonography findings revealed a large 13.6-cm lobulated cystic mass with low-level sonographic echogenicity in the left adnexal region containing a 6-cm solid area with tiny cystic spaces. She underwent an emergency laparotomy for left ovarian cystectomy, with peritoneal washings aspirated for cytological evaluation. Histological diagnosis of the ovarian cyst revealed an isolated benign ovarian leiomyoma within an endometriotic cyst.

## Introduction

Ovarian endometriotic cysts are common findings in gynecology. We are reporting a rare case of an ovarian endometriotic cyst that contained a nodule composed of smooth muscle cells with features of a typical isolated leiomyoma.

It is postulated that ovarian leiomyoma arises from smooth muscle cells in the ovarian hilar blood vessels [1]. The ovarian ligament, smooth muscle cells or multipotential cells in the ovarian stroma, undifferentiated germ cells, or cortical smooth muscle metaplasia are other possible sites of origin [2-4]. Smooth muscle metaplasia of the endometriotic stroma could possibly explain the association seen in this case and in other case reports [5]. Although Fukunga estimates that the incidence of smooth muscle metaplasia in ovarian endometriosis is about 18% [6], there has only been one other report of a leiomyoma clearly arising from endometriosis.

We present a case of an ovarian leiomyoma within an endometriotic cyst in a patient who presented with an acute abdomen, necessitating emergency laparotomy. To our knowledge, there is only one other reported case of a primary ovarian leiomyoma within an endometriotic cyst.

## Case presentation

A 36-year-old nulliparous, healthy woman was admitted to the Singapore General Hospital with a one-day history of intermittent acute lower abdominal pain. The onset of her pain was unrelated to her menstrual cycle or vaginal bleeding. Her last menstrual period was three weeks prior to presentation. She denied any prior unprotected intercourse or possible pregnancy. There were no other urological, gastrointestinal, or constitutional symptoms such as fever, vomiting, diarrhea, or dysuria. She had no significant past medical, surgical, or gynecological history. Her periods have been regular, and the menstrual flow has also been normal, lasting five days every cycle. There was no notable history of dysmenorrhea nor unusual abdominal pains thus far. At the emergency department, physical examination revealed a palpable pelvic mass up to the level of the umbilicus (equivalent to a 20-week gestational size) and an acute abdominal presentation with generalized tenderness and abdominal guarding over the suprapubic and left lower abdominal regions. Her vital signs were stable: temperature 36.6°C, heart rate 89 beats per minute, respiratory rate 19, SpO₂ 99% on room air, and blood pressure 107/72 mmHg. She reported a pain score of eight to nine out of 10 on the visual analogue scale.

**Figure 1:**
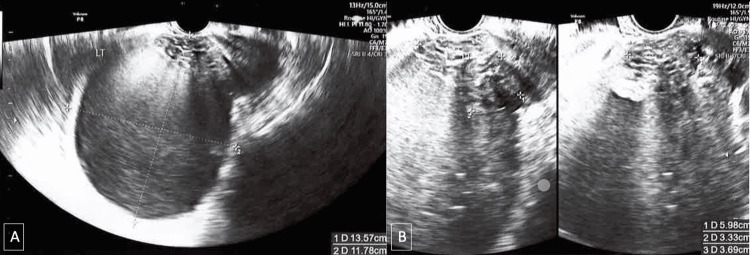
(A) Longitudinal section of left endometriotic cyst. (B) Solid component within left ovarian cyst.

An urgent transvaginal ultrasonography was conducted, which demonstrated a large 13.6 × 11.8 × 13.0 cm lobulated cystic mass with low-level echoes in the left adnexal region containing a 6.0 × 3.3 × 3.7 cm solid area with tiny cystic spaces (Figure 1). Echogenic areas with posterior shadowing were present. No ascites or free fluid was visualized in the pouch of Douglas. The color score of this mass was three, and the likelihood ratio of malignancy of this mass was 6.9% based on the International Ovarian Tumor Analysis (IOTA) score [7]. The uterus appeared normal on the ultrasound.

Her serum cancer antigen 125 was elevated at 46.6 U/mL, and her serum carcinoembryonic antigen and alpha-fetal protein were within normal limits. Her serum human chorionic gonadotropin was negative for pregnancy. Relevant blood investigations are summarized in Table 1.

**Table 1:** Patient’s blood parameters on admission

Laboratory Test	Patient’s Result	Reference Range
Cancer antigen 125	46.6	< 35.1 U/mL
Carcinoembryonic antigen	2	< 4.8 μg/L
Alpha-fetal protein	< 2.7	< 7.1 μg/L
Human chorionic gonadotropin	< 0.6	< 5.3 U/L
Haemoglobin	11.8	12-16 g/dL
White cell count	5.18	4-10 x 10^9^/L
Platelet count	393	140-440 x 10^9^/L

A detailed discussion of further management was conducted. In the context of a raised cancer antigen 125 and the features of the cyst, a frozen section of the cyst will usually be performed to decide on the extent of surgery. However, this service was not available after office hours. She was counselled that, should the final histology reveal malignancy, a second surgery might be necessary. Due to acute pain and concern for ovarian torsion or rupture leading to necrosis and compromised ovarian reserve, she consented to emergency surgery.

**Figure 2:**
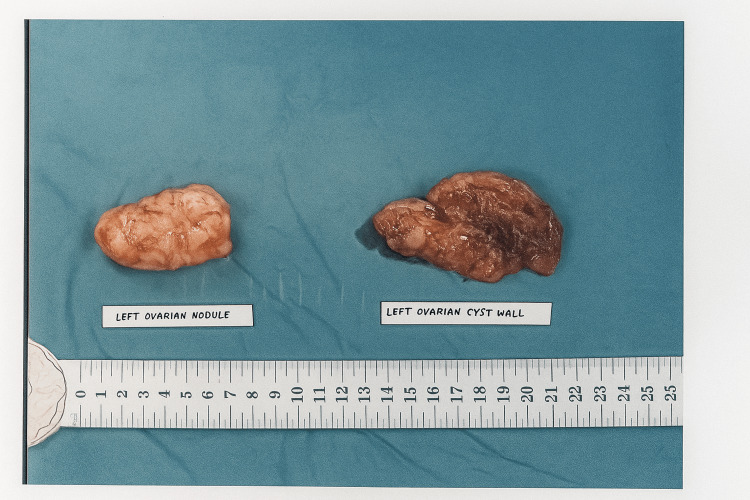
Intraoperative images of left ovarian cyst.

She underwent an emergency laparotomy and left ovarian cystectomy. Intraoperatively, the left ovary contained a 14 cm cyst with hemorrhagic “chocolate” fluid and a distinct 6.5 cm firm, whitish nodular area resembling a fibroma (Figure 2). The left ovary was adherent posteriorly to the bowel with adhesions to the anterior uterus and left ovary. The contralateral right ovary was normal. There were multiple small subserosal fibroids over the uterine fundus and a small amount of ascites in the pelvis. Both fallopian tubes were distended, suggestive of bilateral hydrosalpinx. However, as this was not identified preoperatively and appropriate consent had not been obtained, no surgical intervention was performed. Instead, the patient was managed conservatively with postoperative antibiotics. The entire ovarian cyst was excised intact, and the specimen was later delivered for histological examination. Peritoneal washings were also obtained for cytologic evaluation. The postoperative course was uneventful, and the patient was discharged well on postoperative day two.

**Figure 3:**
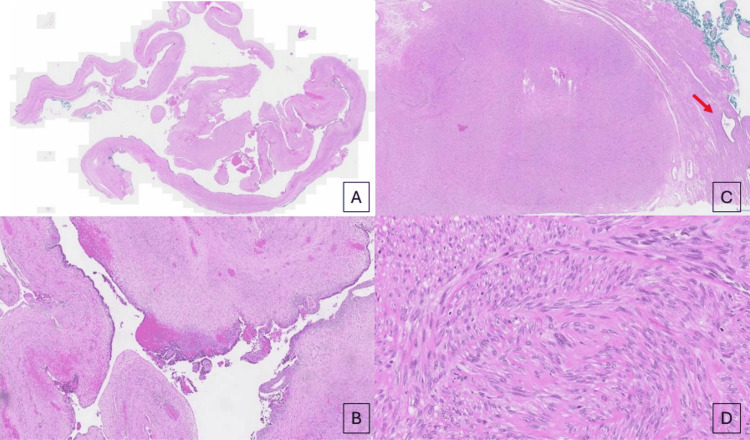
(A,B) Microscopic features of the left ovarian cyst wall. (A) Scanning power image of endometriotic cyst wall and (B) endometrial lining epithelium with underlying endometriotic stroma at higher power. (C,D) Microscopic features of the left ovarian nodule. (C) Low power image of a benign leiomyoma with a focus of endometriosis (red arrow) in the surrounding tissue, and (D) fascicles of smooth muscle tissue confirming the leiomyoma at higher power.

Histological examination revealed the solid component to be an isolated benign ovarian leiomyoma within a benign endometriotic cyst. Histologically, the nodule displayed fascicles of bland spindled smooth muscle cells with eosinophilic and fibrillary cytoplasm, with ovoid elongated nuclei, and with areas of interstitial fibrohyalinization, focal hemorrhage, edema, and variable cellularity (Figure 3). Immunohistochemical staining for desmin and caldesmon was performed on the ovarian nodule, confirming the diagnosis of leiomyoma. The surface of the nodule showed foci of endometriosis. Cytological evaluation of peritoneal washings showed no malignant cells.

## Discussion

Ovarian leiomyoma is often associated with other ovarian lesions, but there has only been one other reported case of a primary ovarian leiomyoma associated with an endometriotic cyst.

Primary ovarian leiomyoma associated with an endometriotic cyst is an extremely rare finding, and only one other case has been reported thus far. It was first reported in 2009 by Tomas et al [8]. There have been several reports of smooth muscle metaplasia within ovarian endometriosis [6], but no other cases of leiomyomas arising from endometriosis have been reported. Smooth muscle metaplasia within ovarian endometriosis is not uncommon, and Fukaunaga estimated that its prevalence was about 18% [6]. Other smooth muscle tumors, such as ovarian adenomyoma associated with an endometriotic cyst, have been previously described by McDougal and Roth [5].

Ovarian leiomyoma is a distinctly unusual tumor of the ovary, as in contrast to the uterus and its supporting ligaments, the ovary itself does not contain smooth muscle cells [9]. It is often an incidental finding during routine pelvic examination, surgery, or after surgical excision of the ovary [10]. It is often difficult to differentiate it from subserosal leiomyomas and ovarian fibromas, and other solid tumors of the ovary until histological analysis is performed [11]. Ovarian leiomyomas were first described by Sangalli in 1862 (cited by Seinera et al.) [12]. Beck et al. estimated that ovarian leiomyomas made up approximately 1% of benign solid ovarian tumors [13]. However, it is likely that many cases remain unreported or misdiagnosed as an ovarian fibroma.

Unlike our case, where the size of the tumor was relatively large and palpable through the abdominal wall, ovarian leiomyomas are generally small and less than a few centimeters in diameter [1]. The tumor typically presents in women between the ages of 20 and 80, with most cases reported in the pre-menopausal population [14,15].

Unilateral ovarian leiomyomas have been predominantly described in case reports, while bilateral ovarian leiomyomas are more frequently reported in young adults and pediatric cases [1]. This is consistent with our patient, who was 36 years old at the time of diagnosis of a unilateral ovarian leiomyoma. Ovarian leiomyomas often co-exist with uterine leiomyomas [16], which is again consistent with the presentation of our patient.

Current literature reports that most women with ovarian leiomyomas are nulliparous. As such, estrogen may play a part in the development of ovarian leiomyomas. Some studies have also suggested the possibility of tumors arising from developmentally abnormal ovaries [17].

Clinically, many patients with ovarian leiomyomas are asymptomatic with no menstrual irregularities. In rapidly growing tumors, weight gain, ascites, and increased abdominal girth can be present [18]. Larger ovarian leiomyomas can also be associated with a raised tumor marker, cancer antigen 125 [19]. It is uncommon that these tumors twist around their pedicle, with hemorrhage and resultant necrosis [18], unlike the mature teratoma. In our case, the patient’s initial presentation with acute abdominal pain may have been related to endometriosis or pelvic infection, the latter suggested by the presence of hydrosalpinx.

On gross examination, these tumors are typically solid and firm with smooth surfaces [20]. The cut-section is typically greyish-white in color, and a whorl formation can be grossly recognizable [20]. Secondary changes, including hyalinization, hemorrhage, calcification, edematous inhibition, and cystic changes, are more frequently observed in larger tumors [20]. A definitive diagnosis of ovarian leiomyoma requires immunohistochemical analysis. Diffuse strong positivity for Smooth Muscle Actin, helping to differentiate it from other fibromatous tumors such as thecoma and fibroma [1,18]. In this case, the leiomyoma was of ovarian origin and formed part of a larger endometriotic cyst.

Radical surgeries are usually performed for complete excision due to diagnostic uncertainty and resemblance to solid pelvic masses [19]. In our present case, we decided to perform an isolated ovarian cystectomy as the mass was clearly and easily separable from the ovary, and the macroscopic appearances looked benign. Given the patient's nulliparous status and reproductive age, fertility-preserving surgery was prioritized.

## Conclusions

In conclusion, leiomyomas are uncommonly found in the ovarian tissue, especially within a background of endometriosis of the ovary. As the clinical manifestation may vary with no consistent sonographic or biochemical distinctions, a high index of suspicion must be exercised, particularly in women of the reproductive age group. Histological diagnosis remains the cornerstone of this unusual pathology.
